# A Quantitative Assessment of MRI-Visible Perivascular-Space Burden Across the Cognitive Spectrum: Associations with APOE ε4 Status and Venous Sinus Volume

**DOI:** 10.3390/diagnostics16152366

**Published:** 2026-07-28

**Authors:** Gvido Kārlis Skuburs, Kristīne Šneidere-Pītersa, Ardis Platkājis, Kalvis Kalva, Zane Anna Litauniece, Agnese Usacka, Zigmunds Freibergs, Gustavs Jānis Bērziņš, Linda Gailite, Aleksejs Isakovs, Ainārs Stepens, Nauris Zdanovskis

**Affiliations:** 1 Department of Radiology, Riga Stradiņš University, Dzirciema Street 16, LV-1007 Riga, Latvia; gvido.skuburs@gmail.com (G.K.S.); ardis.platkajis@rsu.lv (A.P.); gustavs@rsu.edu.lv (G.J.B.); 2 Department of Health Psychology and Paedagogy, Riga Stradiņš University, LV-1007 Riga, Latvia; kristine.sneidere@rsu.lv (K.Š.-P.); agnese.usacka@rsu.lv (A.U.); 3 Institute of Public Health, Riga Stradiņš University, Dzirciema Iela 16, LV-1007 Riga, Latvia; zaneanna.litauniece@rsu.lv (Z.A.L.); zigmunds.freibergs@rsu.lv (Z.F.); ainars.stepens@rsu.lv (A.S.); 4 Institute of Oncology and Molecular Genetics, Riga Stradiņš University, Dzirciema Street 16, LV-1007 Riga, Latvia; linda.gailite@rsu.lv (L.G.); aleksejs.isakovs@rsu.lv (A.I.); 5 Department of Radiology, Riga East University Hospital, Hipokrata Iela 2, LV-1038 Riga, Latvia

**Keywords:** perivascular spaces, glymphatic system, venous sinus volume, cognitive impairment, automated segmentation, dementia, dorsal sagittal sinus, APOE ε4, quantitative neuroimaging biomarkers

## Abstract

**Background/Objectives:** Perivascular spaces (PVS) are MRI-visible compartments surrounding cerebral vessels and are increasingly interpreted as markers of cerebral small-vessel disease and glymphatic-system dysfunction. PVS burden is multifactorial, with possible contributions from vascular risk, atrophy, white matter disease, sleep, inflammation, diabetes, pathological protein deposition, genetic susceptibility, and venous drainage anatomy. This study evaluated whether apolipoprotein E (APOE) ε4 carrier status and venous sinus volumes are associated with quantitative PVS burden in patients across the cognitive spectrum. **Methods**: In this cross-sectional observational MRI study, 110 participants with normal cognition or varying cognitive impairment underwent 3T brain MRI and cognitive assessment. PVS lesion count and total PVS volume were quantified from T2-weighted (T2W) imaging using an automated deep learning segmentation approach. Venous sinus volumes were derived for the transverse, straight, and superior sagittal sinus segments and reported in mm^3^. APOE genotype was analyzed as ε4 carrier status. Non-parametric correlations, ordered cognitive-stage trend tests, APOE group comparisons, and adjusted partial Spearman analyses were performed. **Results**: Dorsal superior sagittal sinus (SSS-D) volume was associated with PVS count (ρ = 0.353, *p* < 0.001) and total PVS volume (ρ = 0.285, *p* = 0.009). These associations persisted after adjustment for age, sex, medial temporal atrophy (MTA), Fazekas score, and estimated intracranial volume (eTIV). APOE ε4 carrier status was not associated with PVS volume or count. PVS burden tended to decrease with greater cognitive-stage severity, while MTA increased across cognitive stages. **Conclusions**: In this cohort, SSS-D volume was associated with automated 3T T2W MRI-detectable PVS burden, whereas APOE ε4 carrier status was not a dominant predictor. These findings support further study of venous sinus morphology as a potential contributor to MRI-visible PVS burden, while longitudinal and flow-sensitive studies are needed to clarify causality and glymphatic clearance mechanisms.

## 1. Introduction

Perivascular spaces (PVS), historically referred to as Virchow–Robin spaces, are fluid-filled compartments that accompany penetrating cerebral vessels [1,2]. They are increasingly discussed in relation to the glymphatic system, a brain fluid-transport pathway implicated in the removal of metabolites and pathological proteins [3,4,5]. Enlarged or highly visible PVS (EPVS) on MRI have been associated with cerebral small-vessel disease, neurodegeneration, and cognitive impairment, although their interpretation is not always straightforward because PVS visibility may vary by anatomical region, disease stage, tissue atrophy, imaging sequence, and segmentation method [6,7,8,9,10,11].

Previous work has shown that PVS burden is related to cognitive and vascular imaging markers; that PVS dilation and white matter hyperintensities (WMH) may serve as neuroimaging biomarkers in normal cognition, mild cognitive impairment, and dementia; and emphasized the value of combined PVS-WMH assessment [10,12,13,14]. Other studies have linked regional PVS volume or EPVS burden to cognition in neurodegenerative disease cohorts, supporting the broader concept that PVS alterations may reflect clinically relevant microvascular or glymphatic dysfunction [13,14,15].

The mechanisms underlying PVS enlargement are likely multifactorial. Reported contributors include hypertension, cerebral atrophy, WMH, sleep disturbance, neuroinflammation, diabetes, and pathological protein deposition [16,17,18]. Genetic susceptibility is also biologically plausible. APOE ε4 is a major genetic risk factor for Alzheimer’s disease and cerebral amyloid angiopathy, and population-based work has reported a modest association between APOE ε4 and high centrum semiovale EPVS burden [19]. APOE may also influence PVS-relevant pathways through blood–brain barrier integrity, cerebrovascular regulation, and amyloid beta clearance [20,21,22,23,24]. However, APOE-PVS associations may be region-specific and may interact with vascular risk factors, making replication in different cohorts and measurement pipelines important.

Venous drainage anatomy is a less frequently studied factor. Because glymphatic and perivascular drainage depend on pressure gradients, vascular pulsatility, and downstream outflow pathways, variation in venous sinus morphology may influence PVS visibility or burden [3,5,16,17,25]. Dural lymphatic and parasagittal drainage studies further support the biological plausibility of considering venous and meningeal outflow anatomy when studying brain fluid clearance [26,27,28,29,30]. Yet venous sinus volumes are not routinely examined together with APOE status and quantitative PVS metrics in cognitive MRI cohorts.

Automated segmentation provides an opportunity to study PVS burden quantitatively rather than relying only on a visual rating scale [31,32]. In this study, PVS were segmented from T2-weighted (T2W) MRI using the T2W nnU-Net workflow from the MedNet-PVS pipeline [32]. Recent studies combining automated EPVS segmentation with DTI-ALPS support the value of quantitative imaging features for evaluating glymphatic-system alterations [15,33]. In the present study, we used automated PVS segmentation and venous sinus volumetry to evaluate whether APOE genotype and venous sinus volumes are associated with automated 3T T2W MRI-detectable PVS count and total volume, and to compare these associations with cognitive and structural imaging markers. Because PVS visibility is sequence-, resolution-, and field-strength-dependent, higher-field (e.g., 7T) MRI may depict additional smaller PVS that are not visible on clinical 3T imaging—therefore, the quantitative PVS measures reported here should be interpreted as automated 3T T2W MRI-detectable PVS burden rather than the complete anatomical PVS compartment. We hypothesized that SSS-D volume would be associated with automated PVS-derived count and total PVS volume after adjustment for demographic and imaging covariates. We also evaluated whether APOE ε4 carrier status was associated with PVS burden.

## 2. Materials and Methods

### 2.1. Study Design and Participants

This cross-sectional observational MRI study included 110 participants. Participant enrollment and imaging were performed between 1 April 2024 and 31 March 2026. Participants were classified clinically into normal cognition, mild cognitive impairment, moderate cognitive impairment, or severe cognitive impairment. For inferential analyses, the moderate and severe groups were combined into a single moderate-to-severe group to improve statistical stability and avoid inference from very small subgroups while preserving the ordered cognitive-severity gradient.

The MoCA-based cognitive impairment categories:

- Normal: 26–30;
- Mild: 11–25;
- Moderate: 6–10;
- Severe: <6.

Ethical Approval and Informed Consent:

This study was conducted in accordance with the Declaration of Helsinki and was approved by the Institutional Review Board of Riga East University Hospital (No. AP-144/10, 3 October 2019) and the Ethics Committee at Riga East University Hospital Ethics Board (No. 08-A/19, 3 October 2019). Written informed consent was obtained from all subjects involved in the study.

### 2.2. MRI Acquisition

All participants underwent 3T brain MRI on the same GE SIGNA Architect scanner (GE HealthCare, Chicago, IL, USA; MR software release DV28.0_R05_2034.a) using the same 48-channel head coil (coil name: 48HAP) and a standardized brain MRI protocol. PVS segmentation was performed on the 3D axial T2W Cube sequence. The key imaging parameters for this sequence were as follows: repetition time (TR) = 2501 ms, echo time (TE) = 157.089 ms, nominal slice thickness = 2.0 mm, reconstructed slice-center spacing = 1.0 mm, field of view = 240 mm, matrix = 512 × 512, reconstructed in-plane pixel spacing = 0.4688 × 0.4688 mm, number of averages = 2, echo train length = 130, and flip angle = 90°. The T2W Cube sequence was acquired as a 3D sequence with 2.0 mm thick reconstructed sections and 1.0 mm spacing between slice centers, corresponding to approximately 50% overlapping reconstructed sections. Therefore, the image series should not be interpreted as true 1.0 mm slice thickness or as having a 1.0 mm interslice gap. No additional manual interpolation or resampling was performed after DICOM export. Imaging covered the basal ganglia (BG), centrum semiovale (CS), and other relevant brain regions.

### 2.3. PVS Segmentation and Quantification

**Figure 1:**
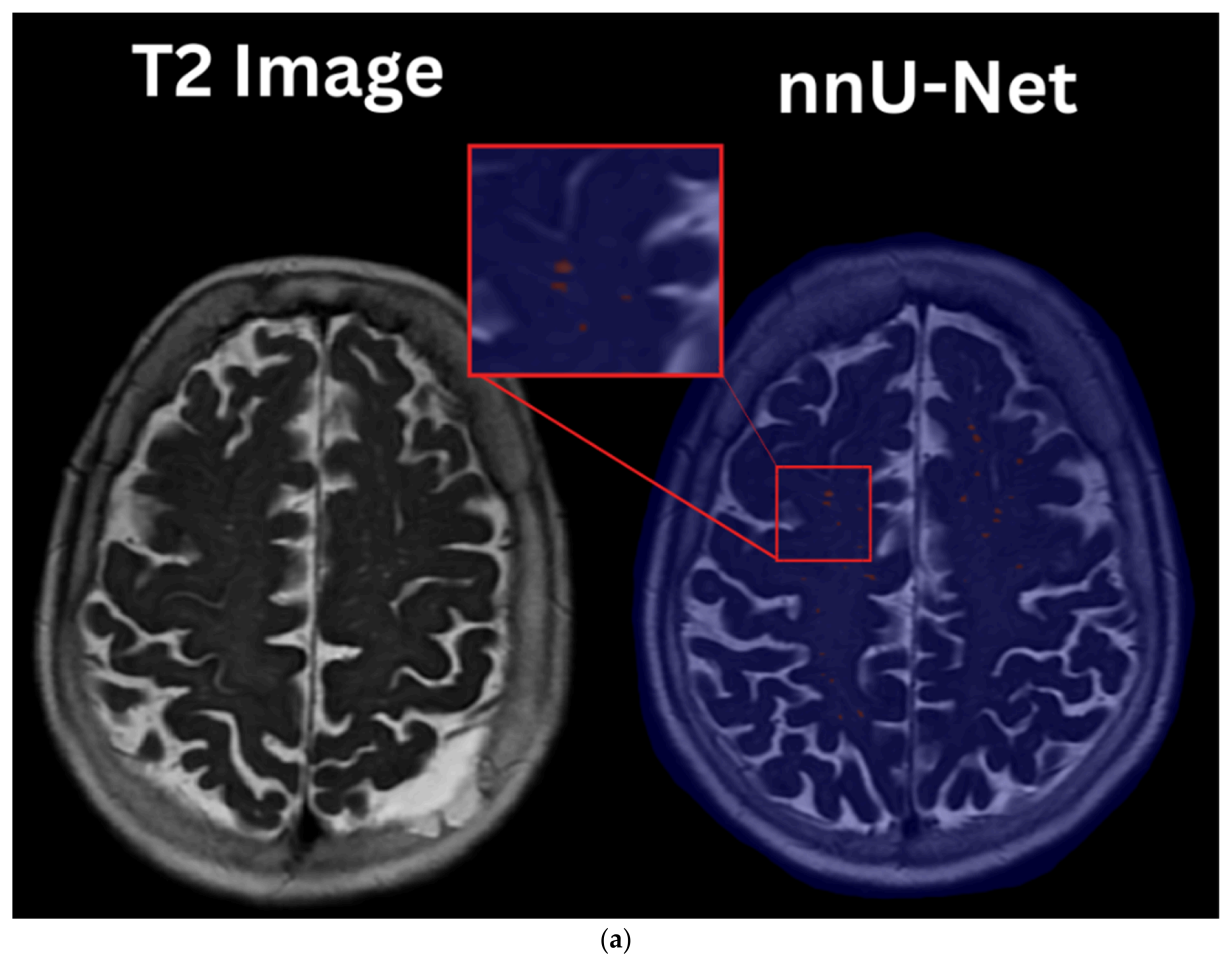

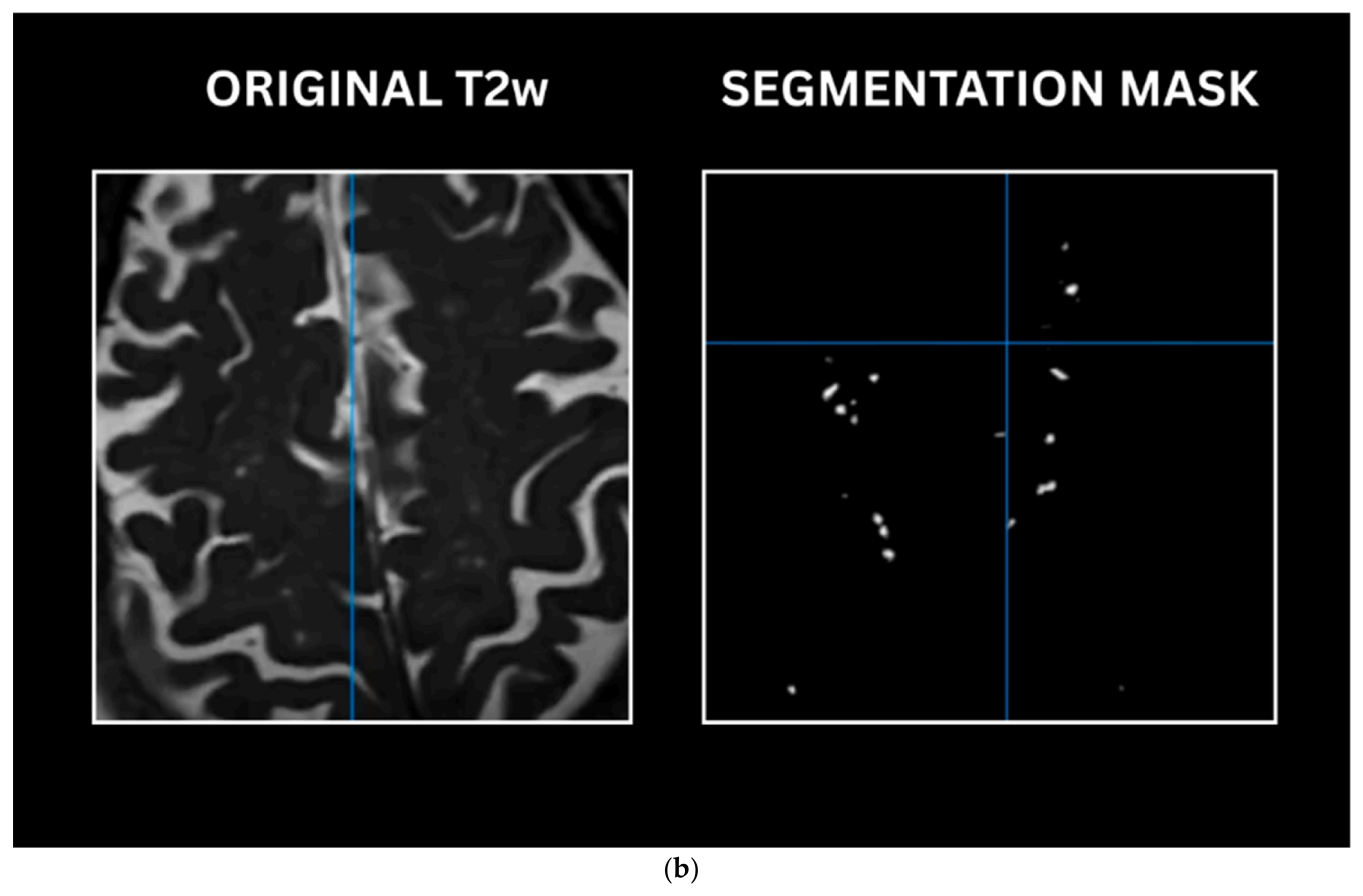
(**a**) A representative example of MRI-visible perivascular-space segmentation; (**b**) the magnified axial T2W MRI input image (**left**) and corresponding automated segmentation mask (**right**). The segmentation model isolated PVS clusters from surrounding brain tissue, enabling the quantitative extraction of lesion count and total PVS volume for statistical analysis.

PVS were segmented using a deep learning approach. Segmentation was performed using the T2W nnU-Net workflow from MedNet-PVS, specifically the 3D full-resolution nnU-Net model trained for T2W images. Preprocessing followed the standard nnU-Net GenericPreprocessor included in the MedNet-PVS workflow. The input image spacing was approximately 1.0 × 0.4688 × 0.4688 mm, and images were internally resampled to 0.8 × 0.8 × 0.8 mm for model inference. No separate z-resampling, custom Otsu-thresholding or zero-filling preprocessing step was applied. The T2W MRI images were used as input for automated segmentation, and the resulting masks were used to extract quantitative PVS metrics. The derived PVS count and volume represent structures detectable under the 3T T2W acquisition and processing conditions. PVS detectability depends on field strength, image contrast, spatial resolution, and segmentation method; for that reason these measurements do not represent the complete anatomical PVS compartment, and smaller PVS detectable at higher field strengths (e.g., 7T) may not be captured. Representative examples of the T2W input image and resulting PVS segmentation output are shown in Figure 1. Quantitative outputs included PVS lesion count, total PVS volume in mL, mean and median lesion volume, maximum segmented PVS object volume, maximum diameter proxy, object-size distribution, and hemispheric asymmetry indices. PVS count was defined as the number of discrete connected segmented PVS objects in the automated binary mask. Total PVS volume was calculated by summing all voxels classified as PVS and multiplying by voxel volume, with conversion to milliliters. In this context, the term “lesion” refers to an individual segmented MRI-visible PVS object or cluster rather than to a destructive tissue lesion. Formal manual quality control, manual correction, and inter-rater validation of the PVS masks were not performed. PVS-derived quantitative measures were reviewed during data processing for completeness and implausible values. PVS volume asymmetry was calculated as (right PVS volume − left PVS volume)/(right PVS volume + left PVS volume). PVS count asymmetry was calculated analogously from right and left lesion counts:

$$PVS\;volume\;asymetry\;index=\frac{Right\;PVS\;volume\;-\;Left\;PVS\;volume}{Right\;PVS\;volume\;+\;Left\;PVS\;volume}$$

The T2W nnU-Net workflow was selected because PVS are CSF-like structures and are more clearly visible on T2W images than on T1-weighted images. In the MedNet-PVS validation study, T2W models showed strong segmentation performance, with voxel-level Dice scores of 0.88 ± 0.06 [27]. T1-weighted performance was lower in the same study. Therefore, the T2W nnU-Net workflow was considered the most appropriate option for the available high-resolution T2W Cube sequence.

Axial T2W MRI image and corresponding automated PVS segmentation output from a representative subject in the cohort. Red voxels indicate segmented enlarged perivascular spaces, illustrating the spatial distribution of PVS burden used for quantitative lesion count and total PVS volume analysis.

### 2.4. Venous Sinus, Genetic, Atrophy and Small-Vessel Disease Variables

Venous sinus measurements were extracted from FreeSurfer’s (v. 8.2.0) mri_vsinus_seg output. This tool segments venous sinuses on the T1-weighted input image, including the left transverse sinus, right transverse sinus, straight sinus, and posterior and dorsal segments of the superior sagittal sinus. Volumetric measures were obtained from the file generated by mri_segstats. These volumes are label-derived physical volumes calculated from the number of voxels assigned to each venous sinus label multiplied by voxel volume. Thus, when voxel dimensions are constant, venous sinus volume is linearly proportional to segmented voxel count. Volume was reported rather than voxel count because it is more anatomically interpretable and consistent with morphometric reporting. Therefore, venous sinus volume refers to the automated FreeSurfer-derived segmented volume of these venous sinus labels rather than to manually traced sinus diameter or flow measurement. These measurements may reflect interindividual variation in venous drainage anatomy, but they were not interpreted as direct measures of venous flow. Volumetric measurements were treated as continuous variables and reported in cubic millimeters. For interpretability in selected figures, venous volumes were also expressed in milliliters, where 1000 mm^3^ equals 1 mL. The posterior and SSS-D were analyzed separately, and total superior sagittal sinus volume was calculated as the sum of the posterior and dorsal segment volumes. Transverse sinus asymmetry was calculated as follows:

$$Transverse\;sinus\;asymmetry\;index=\frac{Right\;transverse\;sinus\;volume\;-\;Left\;tranverse\;sinus\;volume}{Right\;transverse\;sinus\;volume\;+\;Left\;tranverse\;sinus\;volume}$$

Blood samples were collected by a certified specialist for APOE biomarker analysis. APOE genotype data were obtained from the Institute of Oncology and Molecular Genetics, Riga Stradiņš University, based on the APOE-defining single nucleotide variants rs429358 and rs7412. APOE genotype was recorded using predefined dataset codes: 0 = E2/E3, 1 = E3/E3, 2 = E3/E4, 3 = E4/E4, 4 = E2/E4, and 5 = E2/E2. For the main analyses, APOE was analyzed as binary ε4 carrier status. Participants with E3/E4, E4/E4, or E2/E4 genotypes were classified as APOE ε4 carriers, while participants with E2/E3, E3/E3, or E2/E2 genotypes were classified as non-carriers. This grouping was used to compare participants with and without at least one ε4 allele. Participants with the E2/E4 genotype were included in the APOE ε4-carrier group because the primary genetic comparison was based on the presence or absence of at least one ε4 allele. However, E2/E4 participants may not be biologically equivalent to E3/E4 or E4/E4 participants, because ε2 and ε4 alleles can have different or opposing associations with Alzheimer’s disease risk and vascular pathology.

Medial temporal atrophy was recorded separately for the left and right hemispheres and summarized as the mean of the left and right MTA scores. Global cortical atrophy (GCA) was rated manually using a visual GCA scale that considers global brain volume loss, sulcal widening, and cortical thinning. White matter disease burden was represented by the Fazekas score. MTA and GCA were assessed primarily on T1-weighted images, while Fazekas score was assessed on FLAIR images. Visual ratings followed established clinical rating approaches for MTA, GCA, and WMH burden [34,35,36]. MTA, GCA, and Fazekas ratings were performed manually by a board-certified radiologist with specialization in neuroradiology and cognitive impairment. The rater was blinded to APOE genotype and automated PVS-derived quantitative outputs. These variables were used as covariates in adjusted association analyses to evaluate whether associations between venous sinus volume, APOE ε4 carrier status, and PVS burden were independent of demographic, atrophy-related, and small-vessel disease markers.

### 2.5. Statistical Analysis

All statistical analyses were performed using IBM SPSS Statistics version 31.0 and supplemented by custom scripts for permutation testing, rank-residual partial Spearman analyses, and false discovery rate correction (FDR). Descriptive statistics were used to summarize demographic, clinical, genetic, and imaging variables. Continuous variables were reported as median and interquartile range because several imaging measures, including PVS count and PVS volume, were non-normally distributed. Categorical variables were reported as counts and percentages. Benjamini–Hochberg FDR correction was applied separately within each analysis family rather than globally across all statistical tests.

Cognitive-group differences across cognitive categories were assessed using the Kruskal–Wallis test, and ordered monotonic trends were assessed using the Jonckheere–Terpstra test. Pairwise post hoc comparisons were performed when appropriate. Associations between PVS metrics, venous sinus volumes, MoCA score, atrophy measures, and Fazekas score were evaluated using Spearman rank correlation.

APOE was analyzed as binary APOE ε4 carrier status. Differences between APOE ε4 carriers and non-carriers were assessed using non-parametric group comparisons. Adjusted analyses were performed to evaluate whether SSS-D volume, APOE ε4 carrier status, and cognitive severity were independently associated with PVS burden after controlling for age, sex, MTA, Fazekas score, and estimated total intracranial volume (eTIV). eTIV was obtained and derived from the FreeSurfer’s ‘EstimatedTotalIntraCranialVol’ output and was included as a covariate to normalize for individual differences in head size, given that both PVS and venous sinus volumes were measured and reported as absolute physical volumes. They were not normalized by division by eTIV. PVS count and PVS volume were transformed using the natural logarithm of one plus the value for adjusted analyses because of right-skewed distributions. Multiple-comparison correction was performed using the Benjamini–Hochberg false discovery rate procedure. Statistical significance was set at *p* < 0.05, with FDR-adjusted q < 0.05 considered statistically robust after correction.

### 2.6. Usage of AI Tools

AI tools were used for editing and improving the abstract, including grammar, readability and wording. We reviewed all AI-assisted edits and take full responsibility for the final content.

## 3. Results

### 3.1. Cohort Characteristics After Cognitive-Group Collapse

Cognitive-group analysis included 110 participants with classifiable cognitive status: 41 with normal cognition, 49 with mild impairment, and 20 with moderate-to-severe impairment. Cohort characteristics by cognitive group are shown in Table 1.

Additional descriptive checks were performed to assess demographic balance and left–right asymmetry. Age increased across the collapsed cognitive groups, whereas sex distribution was similar across groups. Age and sex distributions were also similar between APOE ε4 carriers and non-carriers. Exploratory left–right analyses showed minimal PVS asymmetry, with no statistically robust difference between left and right PVS volume or count. The median Fazekas score was 1.00 [1.00, 1.00] in all collapsed cognitive groups, whereas the median mean MTA score increased to 2.00 [1.00, 2.00] in the moderate-to-severe impairment group.

### 3.2. PVS Burden Across Cognitive Groups

PVS burden showed weak inverse trends across cognitive-severity groups. Median PVS count was 172.50 [104.75, 280.50] in the normal cognition group, 124.00 [68.00, 196.50] in the mild impairment group, and 135.00 [39.50, 212.50] in the moderate-to-severe impairment group. Median PVS volume was 1.00 mL [0.62, 2.01], 0.62 mL [0.31, 1.27], and 0.69 mL [0.16, 1.48], respectively.

**Figure 2:**
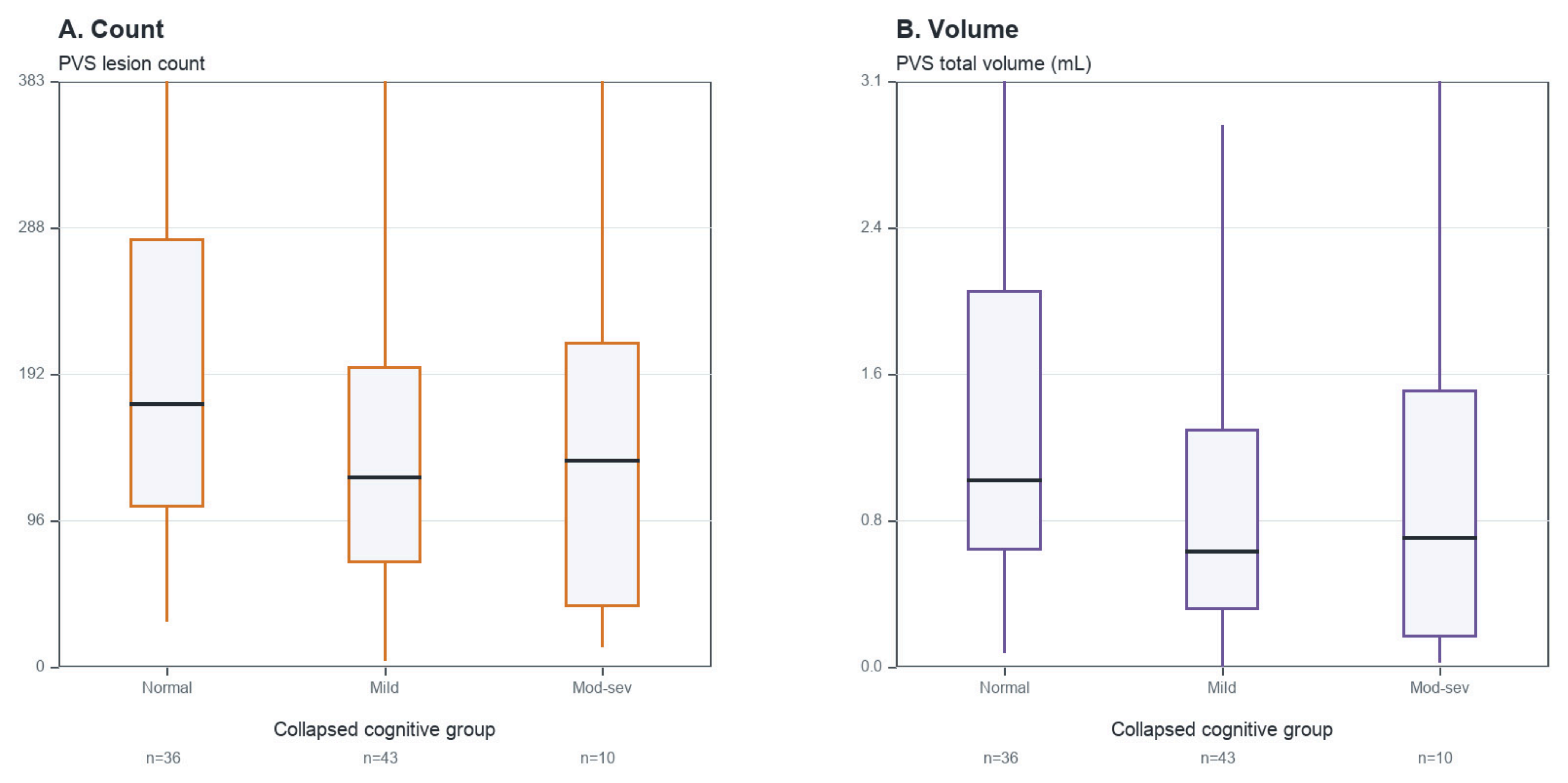
PVS burden across cognitive groups. Box plots summarize (**A**) PVS lesion count and (**B**) total PVS volume across normal cognition, mild impairment, and moderate-to-severe impairment groups.

Jonckheere trend testing suggested decreasing PVS count (*p* = 0.050, FDR q = 0.109) and decreasing PVS volume (*p* = 0.043, FDR q = 0.109) with greater cognitive severity; however, neither association survived FDR correction. Kruskal–Wallis tests similarly did not show robust group differences in PVS count or PVS volume. The distribution of PVS burden across collapsed cognitive groups is shown in Figure 2, and the main cognitive-group tests are summarized in Table 2.

Pairwise tests suggested higher PVS count and PVS volume in the normal cognition group than in the mild impairment group, but these pairwise findings did not survive within-outcome FDR correction. For PVS count, the normal cognition versus mild impairment comparison had *p* = 0.031 and FDR q = 0.093. For PVS volume, the corresponding comparison had *p* = 0.027 and FDR q = 0.081. Pairwise PVS comparisons involving the moderate-to-severe group were not statistically robust.

### 3.3. Venous Sinus Volume and PVS Burden

**Figure 3:**
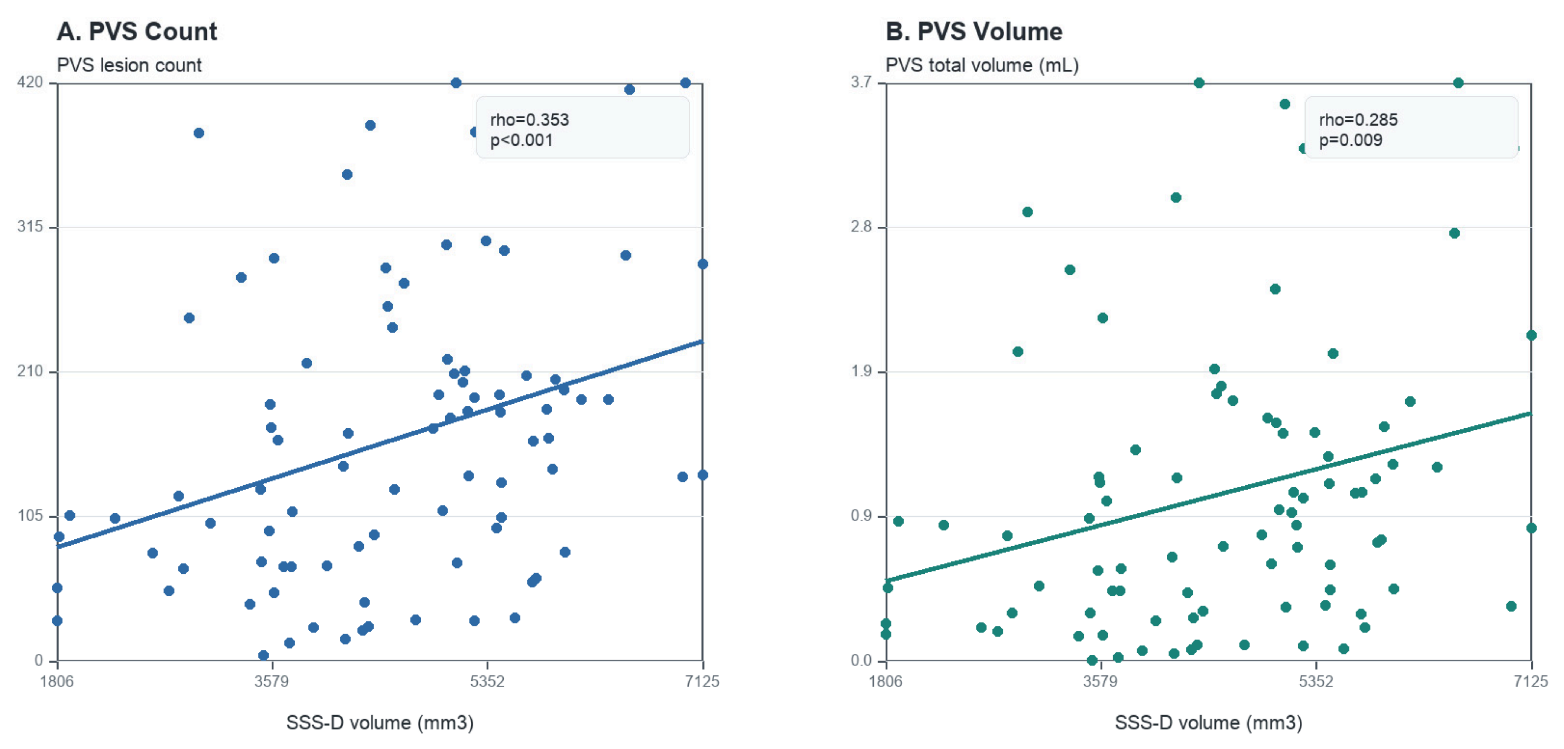
Association between SSS-D volume and automated 3T T2W MRI-detectable PVS burden. Scatterplots show SSS-D volume against (**A**) PVS lesion count and (**B**) total PVS volume. Trend lines are shown for visual orientation; inferential analyses used Spearman rank correlation and adjusted partial Spearman models.

Dorsal superior sagittal sinus volume remained the most consistent imaging correlate of automated PVS-derived burden. In unadjusted analyses, SSS-D volume correlated with PVS count (Spearman rho = 0.353, 95% CI [0.160, 0.519], *p* < 0.001, FDR q = 0.020) and with PVS volume (rho = 0.285, 95% CI [0.077, 0.460], *p* = 0.009, FDR q = 0.058). Dorsal SSS volume did not differ across the three cognitive groups (normal 4760 mm^3^ [3582, 5449]; mild 4397 [3575, 5465]; moderate-to-severe 4885.50 [4083.25, 5300.00]; Kruskal–Wallis *p* = 0.772, FDR q = 0.913; Jonckheere trend *p* = 0.82). Because dorsal SSS volume was associated with PVS burden yet was itself unrelated to cognitive stage, the sinus-PVS association is unlikely to be confounded by cognitive severity. The relationship between SSS-D volume and PVS burden is shown in Figure 3.

To contextualize the relationship between venous sinus anatomy, PVS burden, cognition, and atrophy, we performed Spearman correlation analysis across the core imaging and clinical variables. SSS-D volume was positively associated with PVS count and total PVS volume. In contrast, SSS-D volume was not significantly associated with MoCA, MTA, or GCA after FDR correction. MoCA showed stronger inverse associations with MTA and GCA than with total PVS volume, suggesting that cognitive performance in this cohort was more closely aligned with atrophy measures than with PVS burden alone. These associations are summarized in Table 3. Additional correlations between PVS count, total PVS volume, and PVS object-size metrics are provided in Table S2.

**Figure 4:**
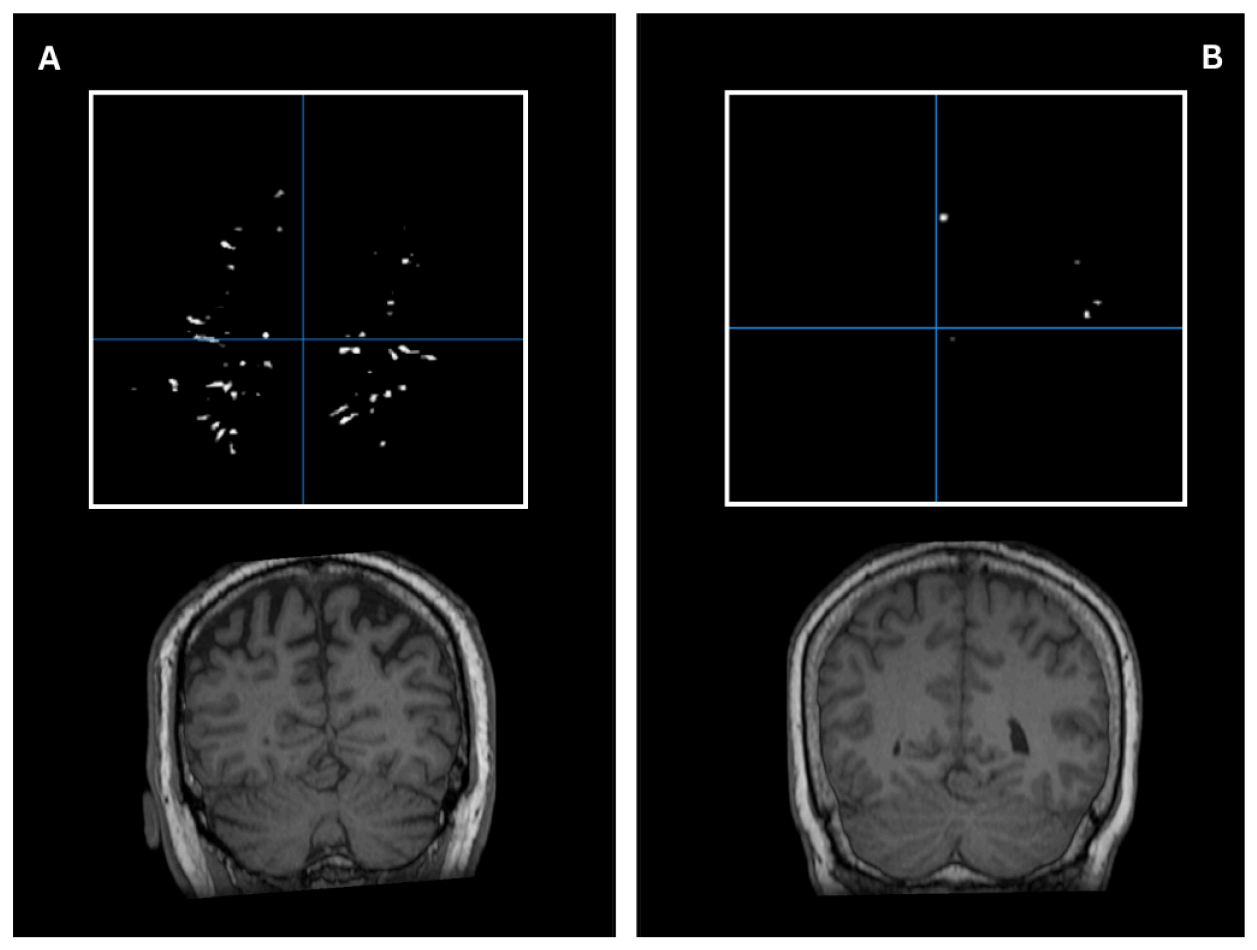
A representative comparison of SSS-D volume and automated 3T T2W MRI-detectable PVS burden. The lower panels show coronal T1-weighted structural MRI images for anatomical orientation only. Quantitative PVS segmentation and PVS metrics were derived from T2W MRI. The upper panels show the corresponding automated PVS segmentation masks. (**A**) A participant with larger dorsal superior sagittal sinus volume (SSS-D volume: 6.52 mL) and higher PVS burden (PVS count: 415; total PVS volume: 4.34 mL). (**B**) A participant with smaller dorsal superior sagittal sinus volume (SSS-D volume: 1.76 mL) and lower PVS burden (PVS count: 29; total PVS volume: 0.17 mL). These examples illustrate the cohort-level association between SSS-D and automated PVS-derived burden.

Representative examples of participants with different SSS-D volumes and corresponding automated PVS-derived burden are shown in Figure 4.

### 3.4. APOE ε4 Carrier Status and PVS Burden

**Figure 5:**
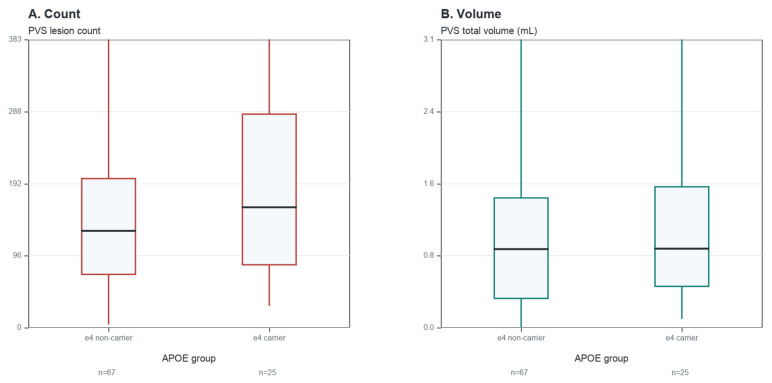
PVS burden by APOE ε4 carrier status. Box plots summarize (**A**) PVS lesion count and (**B**) total PVS volume in APOE ε4 non-carriers and carriers.

APOE was analyzed as a binary variable: APOE ε4 non-carrier versus APOE ε4 carrier. Among participants with PVS measurements, APOE ε4 carrier status was not associated with PVS count or PVS volume. Median PVS count was 129.00 [70.00, 199.00] in non-carriers and 160.00 [83.00, 285.00] in carriers (*p* = 0.226, FDR q = 0.588). Median PVS volume was 0.86 mL [0.31, 1.41] in non-carriers and 0.86 mL [0.44, 1.54] in carriers (*p* = 0.638, FDR q = 0.830). Binary APOE ε4 group comparisons are summarized in Table 4 and shown in Figure 5.

### 3.5. Adjusted Association Analyses

Adjusted partial Spearman analyses were used to evaluate whether collapsed cognitive severity, APOE ε4 carrier status, and SSS-D volume were independently associated with PVS burden. After adjustment for age, sex, MTA, Fazekas score, and eTIV, collapsed cognitive severity was not independently associated with log PVS count (partial rho = −0.134, *p* = 0.228, FDR q = 0.596) or log PVS volume (partial rho = −0.113, *p* = 0.319, FDR q = 0.627). APOE ε4 carrier status was also not independently associated with log PVS count or log PVS volume.

**Figure 6:**
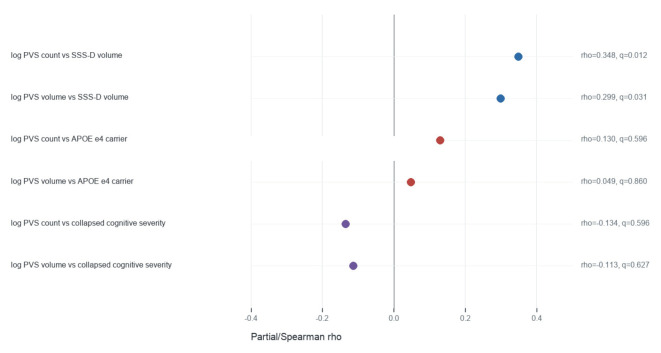
Fully adjusted association summary. The forest-style plot shows partial Spearman estimates for associations between primary predictors and log-transformed PVS outcomes after adjustment for age, sex, MTA, Fazekas score, and eTIV.

In contrast, SSS-D volume remained associated with PVS burden after full adjustment. SSS-D volume was associated with log PVS count (partial rho = 0.348, *p* = 0.001, FDR q = 0.012) and log PVS volume (partial rho = 0.299, *p* = 0.006, FDR q = 0.031) after adjustment for age, sex, MTA, Fazekas score, and eTIV. Adjusted analyses are summarized in Table 5 and visualized in Figure 6.

### 3.6. Summary of Results

For inferential analyses, the original cognitive categories were collapsed into three clinically interpretable groups: normal cognition, mild impairment, and moderate-to-severe impairment. This improved statistical stability while preserving the clinical severity gradient. Age increased across the collapsed cognitive groups, whereas sex distribution was similar across groups. Age and sex distributions were also similar between APOE ε4 carriers and non-carriers (Table S1). The collapsed analysis did not materially change the main result pattern. PVS burden showed weak inverse trends across cognitive-severity groups, but these associations were not FDR-robust and were not independent after covariate adjustment. APOE ε4 carrier status was not associated with PVS count or volume in unadjusted, binary group, or adjusted analyses. Exploratory left–right analyses showed minimal PVS asymmetry. Fazekas scores were similar across collapsed cognitive groups, while mean MTA was highest in the moderate-to-severe group. Overall, SSS-D volume remained the most consistent correlate of automated PVS-derived burden, particularly PVS count, even after adjustment for age, sex, MTA, Fazekas score, and eTIV.

## 4. Discussion

This cross-sectional MRI study evaluated whether APOE genotype and venous sinus anatomy are associated with quantitative automated 3T T2W MRI-detectable PVS burden in participants across the cognitive spectrum. The most consistent finding was a positive association between SSS-D volume and PVS burden. This relationship was observed for PVS count and, to a lesser extent, total PVS volume, and remained present after adjustment for age, sex, MTA, Fazekas score, and eTIV. In contrast, APOE ε4 carrier status was not a dominant predictor of PVS burden in this cohort.

These findings support a multifactorial model of MRI-visible PVS burden. PVS are often interpreted as markers of glymphatic or perivascular drainage dysfunction, but their MRI visibility is influenced by multiple biological and technical factors [2,4,5,6,19]. PVS count and total PVS volume showed weak inverse trends with greater cognitive severity, but these effects did not remain robust after correction for multiple comparisons and were not independent after covariate adjustment. Our weak inverse PVS trend contrasts with larger studies reporting higher PVS burden at greater cognitive severity; this discrepancy may be due to reduced automated PVS conspicuity in atrophied brains lowering detected count and volume, and our trend did not survive multiple-comparison correction (FDR q = 0.109), so we interpret it as non-robust. One plausible explanation is that increasing atrophy in later disease stages may alter the visibility, segmentation, or apparent volume of PVS, rather than reflecting a simple monotonic increase in glymphatic dysfunction. This interpretation is consistent with longitudinal MRI studies using automated image analysis, which suggest that PVS-related risk may depend not only on total PVS volume but also on PVS number, lesion size, white matter lesion burden, brain atrophy, and disease stage [11,12,15]. Because dorsal SSS volume was associated with PVS burden yet was itself unrelated to cognitive stage, the sinus–PVS association is unlikely to be confounded by cognitive severity.

The absence of a strong APOE ε4 association does not exclude a role for APOE in perivascular or glymphatic biology. Previous population-based work has suggested that APOE ε4 may be linked to high centrum semiovale enlarged PVS burden, particularly in the context of small-vessel disease topography and vascular risk [28]. The present study differed in cohort composition, sample size, cognitive enrichment, whole-brain PVS quantification, and available vascular and amyloid biomarker information. APOE ε4 remains mechanistically relevant because it has been associated with blood–brain barrier dysfunction, altered cerebrovascular integrity, impaired neurovascular regulation, and amyloid-beta clearance pathways [20,29,30,31,32,33]. Therefore, the current findings should be interpreted as evidence that APOE ε4 was not a dominant predictor of whole-brain quantitative PVS burden, rather than evidence against any APOE contribution to PVS pathophysiology.

The association between SSS-D volume and PVS burden suggests that venous sinus morphology may be relevant to MRI-visible perivascular-space burden. This finding should not be interpreted as proof of impaired venous outflow or causal glymphatic dysfunction, because venous volume is an anatomical marker and not a direct flow measurement. However, the result is biologically plausible considering studies linking cerebrospinal fluid movement to vascular pulsatility and studies showing drainage toward dural lymphatic and parasagittal pathways [16,17,18,22,23,24,25,26]. These findings identify venous sinus anatomy as a potentially important imaging variable to include in future PVS studies. Although we quantified PVS on T2W imaging because PVS are CSF-like and more conspicuous on T2W than on T1W (in the MedNet-PVS validation, T2W Dice = 0.88 ± 0.06 exceeded T1W performance), the biological rationale linking venous sinus morphology to perivascular clearance is sequence-independent and is for that reason equally relevant to T1-based PVS studies. Because T1- and T2W-derived pipelines detect partly different PVS populations, the quantitative association reported here should not be assumed to transfer unchanged to T1-derived metrics and warrants independent replication in T1-based pipelines. Future studies using phase-contrast MRI, venous flow measures, contrast-enhanced lymphatic imaging, DTI-ALPS, amyloid/tau biomarkers, sleep measures, and longitudinal cognitive follow-up are needed to test this hypothesis directly.

Study limitations. Several limitations should be considered when interpreting these findings. The cross-sectional design limits causal inference and does not allow evaluation of longitudinal changes in PVS burden, venous sinus morphology, atrophy, or cognition. Amyloid and tau biomarkers were not available, so APOE-related associations could not be interpreted in relation to molecular Alzheimer-type pathology. PVS burden was quantified using an automated segmentation pipeline, and quantitative outputs were reviewed for completeness, implausible values, and outliers during data processing and analysis. However, formal manual correction and inter-rater validation of segmentation masks were outside the scope of the present study; therefore, the PVS measures should be interpreted as automated quantitative imaging biomarkers. MTA, global cortical atrophy, and Fazekas scores were visually rated by a certified radiologist, which reflects standard clinical imaging practice but may retain some observer dependence. In more advanced cognitive impairment, cerebral atrophy may also influence the visibility or apparent volume of automated 3T T2W MRI-detectable PVS burden, which could partly explain the weak inverse PVS trend across cognitive groups. Consistent with this, both medial temporal atrophy (Kruskal–Wallis *p* < 0.001) and global cortical atrophy (*p* = 0.037) increased significantly across cognitive groups, whereas eTIV did not differ (*p* = 0.23). Direct brain tissue volumes (e.g., total gray or supratentorial volume) were not available from the segmentation pipeline used here; nonetheless the visual atrophy ratings indicate greater atrophy at higher cognitive severity, consistent with reduced automated PVS conspicuity contributing to the observed inverse trend. FreeSurfer describes mri_vsinus_seg as a tool primarily intended to improve skull stripping and prevent pial surface extension into venous sinuses; it has not been validated as a dedicated method for accurate venous sinus volumetry. Therefore, venous sinus volumes in this study should be interpreted as automated FreeSurfer-derived anatomical estimates. The sample size was moderate, and moderate and severe cognitive impairment categories were combined to improve statistical stability.

Further longitudinal studies with larger cohorts are needed to confirm these findings and clarify the temporal relationship between venous sinus morphology, PVS burden, brain atrophy, and cognitive decline. Future research should also incorporate complementary imaging and biomarker approaches, including phase-contrast MRI, DTI-ALPS, CSF-dynamics assessment, and amyloid and tau biomarkers, to better characterize the mechanisms underlying PVS enlargement and glymphatic dysfunction.

Taken together, the findings support a multifactorial interpretation of automated PVS-derived burden. In this cohort, SSS-D volume showed a stronger and more consistent relationship with quantitative PVS burden than APOE ε4 carrier status. These results support the inclusion of venous sinus morphology in future PVS and glymphatic-imaging studies, while emphasizing the need for longitudinal and flow-sensitive imaging to clarify mechanisms.

## 5. Conclusions

In this cross-sectional observational MRI cohort, dorsal superior sagittal sinus volume was associated with automated 3T T2W MRI-detectable PVS count and volume independently of demographic, atrophy-related, small-vessel disease, and intracranial-volume covariates. APOE ε4 carrier status was not associated with PVS burden. These findings suggest that venous sinus morphology may contribute to quantitative PVS burden or visibility, while APOE genotype was not a dominant determinant in this dataset. Future longitudinal studies incorporating venous flow measures, vascular risk factors, amyloid/tau biomarkers, and glymphatic imaging indices are needed to clarify causal pathways linking venous anatomy, PVS, atrophy, and cognitive decline.

## Figures and Tables

**Table 1:** Cohort characteristics by collapsed cognitive group.

Variable	Normal Cognition	Mild Impairment	Moderate-to-Severe Impairment
Participants, *n*	41	49	20
Age, years	66.00 [61.50, 72.00]	71.00 [66.25, 78.50]	75.00 [67.75, 80.00]
Female sex, *n* (%)	27 (65.9%)	30 (61.2%)	13 (65.0%)
MoCA score	26.00 [25.00, 27.00]	23.00 [20.00, 24.00]	16.00 [13.75, 17.00]
APOE ε4 carrier, *n* (%)	11 (26.8%)	12 (24.5%)	6 (30.0%)
PVS count	172.50 [104.75, 280.50]; *n* = 36	124.00 [68.00, 196.50]; *n* = 43	135.00 [39.50, 212.50]; *n* = 10
PVS volume, mL	1.00 [0.62, 2.01]; *n* = 36	0.62 [0.31, 1.27]; *n* = 43	0.69 [0.16, 1.48]; *n* = 10
SSS-D volume, mm^3^	4760.00 [3582.00, 5449.00]; *n* = 39	4397.00 [3575.00, 5465.00]; *n* = 46	4885.50 [4083.25, 5300.00]; *n* = 20
MTA mean	1.00 [1.00, 1.00]	1.00 [0.00, 1.00]	2.00 [1.00, 2.00]
Fazekas score	1.00 [1.00, 1.00]	1.00 [1.00, 1.00]	1.00 [1.00, 1.00]

Note: Values are median [IQR] unless otherwise specified. PVS = perivascular spaces; SSS-D = dorsal superior sagittal sinus; MoCA = Montreal Cognitive Assessment; MTA = medial temporal atrophy.

**Table 2:** Main collapsed cognitive-group tests.

Outcome	*n*	Jonckheere Direction	Jonckheere *p*	Jonckheere FDR q	Kruskal–Wallis H	Kruskal–Wallis *p*	Kruskal–Wallis FDR q
PVS count	89	decreases with severity	0.05	0.109	4.791	0.096	0.208
PVS volume	89	decreases with severity	0.043	0.109	4.906	0.085	0.208
SSS-D volume	105	increases with severity	0.82	0.945	0.538	0.772	0.913
MTA mean	109	increases with severity	0.001	0.008	16.17	<0.001	<0.001
Fazekas	108	increases with severity	0.264	0.429	1.016	0.504	0.776
MoCA	110	decreases with severity	<0.001	0.001	77.477	<0.001	<0.001
APOE ε4 carrier	110	increases with severity	0.945	0.945	0.132	0.885	0.958

Note: Jonckheere trend tests evaluate ordered monotonic trends across the three cognitive groups. Kruskal–Wallis tests evaluate overall distributional differences across groups. FDR q-values use Benjamini–Hochberg correction.

**Table 3:** Spearman correlation matrix of core venous, PVS, cognitive, and atrophy metrics.

Variable	SSS-D Volume	PVS Count	PVS Volume	MoCA	MTA	GCA
SSS-D volume	-	0.355 (q = 0.005)	0.289 (q = 0.020)	−0.083 (q = 0.520)	0.081 (q = 0.520)	0.186 (q = 0.096)
PVS count	0.355 (q = 0.005)	-	0.972 (q = <0.001)	0.177 (q = 0.157)	−0.249 (q = 0.041)	−0.095 (q = 0.520)
PVS volume	0.289 (q = 0.020)	0.972 (q = <0.001)	-	0.211 (q = 0.087)	−0.258 (q = 0.034)	−0.083 (q = 0.520)
MoCA	−0.083 (q = 0.520)	0.177 (q = 0.157)	0.211 (q = 0.087)	-	−0.321 (q = 0.004)	−0.224 (q = 0.041)
MTA	0.081 (q = 0.520)	−0.249 (q = 0.041)	−0.258 (q = 0.034)	−0.321 (q = 0.004)	-	0.565 (q < 0.001)
GCA	0.186 (q = 0.096)	−0.095 (q = 0.520)	−0.083 (q = 0.520)	−0.224 (q = 0.041)	0.565 (q =< 0.001)	-

Note: Cells show Spearman rho with Benjamini–Hochberg FDR-corrected q-values in parentheses. SSS-D = dorsal superior sagittal sinus; PVS = perivascular spaces; MoCA = Montreal Cognitive Assessment; MTA = medial temporal atrophy; GCA = global cortical atrophy.

**Table 4:** Binary APOE ε4 group comparisons.

Outcome	APOE ε4 Non-Carrier, Median [IQR]	APOE ε4 Carrier, Median [IQR]	*n* Non-Carrier	*n* Carrier	Rank-Biserial r (95% CI)	*p*	FDR q
PVS count	129.00 [70.00, 199.00]	160.00 [83.00, 285.00]	67	25	−0.165 [−0.422, 0.097]	0.226	0.588
PVS volume, mL	0.86 [0.31, 1.41]	0.86 [0.44, 1.54]	67	25	−0.066 [−0.323, 0.195]	0.638	0.83
SSS-D volume, mm3	4372.00 [3558.00, 5191.00]	5247.00 [4409.50, 5701.50]	81	27	-	0.008	0.107
SSS total volume, mm3	7768.00 [6285.00, 8796.00]	8582.00 [6858.00, 9765.50]	81	27	-	0.016	0.107
MoCA	24.00 [20.00, 26.00]	23.00 [18.00, 25.00]	83	29	-	0.449	0.83
Collapsed cognitive severity	1.00 [0.00, 1.00]	1.00 [0.00, 2.00]	81	29	-	0.941	0.979

Note: *p*-values are from two-group rank-based permutation tests. FDR q-values are corrected across binary APOE comparisons. Rank-biserial r values with bootstrap 95% confidence intervals are shown for the primary APOE-PVS comparisons. SSS-D and SSS total volume were numerically larger in APOE ε4 carriers, but these venous comparisons did not survive FDR correction.

**Table 5:** Fully adjusted partial Spearman analyses for primary PVS outcomes.

Outcome	Predictor	Covariates	*n*	Partial Spearman Rho	95% CI	*p*	FDR q
log PVS count	Collapsed cognitive severity	age + sex + MTA + Fazekas + eTIV	81	−0.134	[−0.383, 0.146]	0.228	0.596
log PVS volume	Collapsed cognitive severity	age + sex + MTA + Fazekas + eTIV	81	−0.113	[−0.363, 0.176]	0.319	0.627
log PVS count	APOE ε4 carrier	age + sex + MTA + Fazekas + eTIV	83	0.13	[−0.110, 0.355]	0.239	0.596
log PVS volume	APOE ε4 carrier	age + sex + MTA + Fazekas + eTIV	83	0.049	[−0.179, 0.281]	0.669	0.86
log PVS count	SSS-D volume	age + sex + MTA + Fazekas + eTIV	83	0.348	[0.151, 0.525]	0.001	0.012
log PVS volume	SSS-D volume	age + sex + MTA + Fazekas + eTIV	83	0.299	[0.083, 0.492]	0.006	0.031

Note: Partial Spearman analyses used rank residuals after covariate adjustment. eTIV = estimated total intracranial volume. Confidence intervals are bootstrap 95% confidence intervals based on 5000 resamples.

## Data Availability

The data presented in this study are available on request from the corresponding author due to privacy and ethical restrictions related to human MRI, cognitive, and genetic data.

## Supplementary Materials

The following supporting information can be downloaded at: https://www.mdpi.com/article/10.3390/diagnostics16152366/s1.

## Abbreviations

The following abbreviations are used in this manuscript:

MRI	Magnetic resonance imaging
T1W	T1-weighted
T2W	T2-weighted
PVS	Perivascular spaces
WMH	White matter hyperintensities
MoCA	Montreal Cognitive Assessment
MTA	Medial temporal atrophy
FDR	False discovery rate
SSS-D	Dorsal superior sagittal sinus
GCA	Global cortical atrophy
eTIV	Estimated total intracranial volume
DTI-ALPS	Diffusion tensor image analysis along the perivascular space
FLAIR	Fluid-attenuated inversion recovery
APOE	Apolipoprotein E
