# Supplementary material for: A Quantitative Assessment of MRI-Visible Perivascular-Space Burden Across the Cognitive Spectrum: Associations with APOE ε4 Status and Venous Sinus Volume
Source: Diagnostics (Basel). 2026 Jul 28;16(15):2366. doi: 10.3390/diagnostics16152366 (PMC13463903; doi:10.3390/diagnostics16152366)
Supplement: Supplementary file 1 [file diagnostics-16-02366-s001.zip › diagnostics-4351733-supplementary.pdf]

Supplementary Materials

Diagnostics 2026 - Quantitative Assessment of MRI-Visible Perivascular Space Burden Across the Cognitive Spectrum: Associations with APOE ε4 Status and Venous Sinus Volume

Table S1. Demographic, cognitive, and imaging characteristics stratified by cognitive group and APOE ε4 carrier status.

Panel A. By cognitive group

Variable	Normal cognition (n = 41)	Mild impairment (n = 49)	Moderate-to-severe (n = 20)	p-value
Age, years	66.00 [61.50, 72.00]	71.00 [66.25, 78.50]	75.00 [67.75, 80.00]	0.001[a]
Female sex, n (%)	27 (65.9%)	30 (61.2%)	13 (65.0%)	0.893[b]
APOE ε4 carrier, n (%)	11 (26.8%)	12 (24.5%)	6 (30.0%)	0.892[b]
MoCA score	26.00 [25.00, 27.00]	23.00 [20.00, 24.00]	16.00 [13.75, 17.00]	< 0.001[a]
PVS count	172.50 [104.75, 280.50]	124.00 [68.00, 196.50]	135.00 [39.50, 212.50]	0.096[a]
PVS volume, mL	1.00 [0.62, 2.01]	0.62 [0.31, 1.27]	0.69 [0.16, 1.48]	0.085[a]
SSS-D volume, mm^3	4760.00 [3582.00, 5449.00]	4397.00 [3575.00, 5465.00]	4885.50 [4083.25, 5300.00]	0.772[a]
MTA mean score	1.00 [1.00, 1.00]	1.00 [0.00, 1.00]	2.00 [1.00, 2.00]	< 0.001[a]
Fazekas score	1.00 [1.00, 1.00]	1.00 [1.00, 1.00]	1.00 [1.00, 1.00]	0.504[a]

Panel B. By APOE ε4 carrier status

Variable	APOE ε4 non-carrier	APOE ε4 carrier	p-value
Participants, n	81	29	—
Age, years	68.50 [65.00, 75.00]; n = 78	71.00 [66.50, 76.50]; n = 27	0.766[c]
Female sex, n (%)	52 (64.2%)	18 (62.1%)	0.838[b]

Values are median [interquartile range] unless otherwise specified. A Kruskal–Wallis test; b Pearson χ^2 test; c Mann–Whitney U test. No statistically significant differences in sex or APOE ε4 carrier distribution were observed across cognitive groups, and age and sex did not differ between APOE ε4 carriers and non-carriers. Age increased significantly across cognitive groups (p = 0.001). PVS = perivascular spaces; SSS-D = dorsal superior sagittal sinus; MoCA = Montreal Cognitive Assessment; MTA = medial temporal atrophy.

Table S2. Spearman correlations among PVS count, total PVS volume, and PVS object-size metrics.

Comparison	Spearman ρ	p-value	n
PVS count vs total PVS volume	0.972	< 0.001	92
Mean PVS object volume vs total PVS volume	0.818	< 0.001	92
Maximum PVS object volume vs total PVS volume	0.842	< 0.001	92

Spearman rank correlations computed on participants with available automated PVS quantification. Although PVS count and total PVS volume were strongly correlated, they are not identical measures: PVS count reflects the number of segmented PVS objects, whereas total PVS volume reflects both object number and object size. Consequently, associations with predictor variables may differ slightly between count-based and volume-based PVS outcomes. PVS = perivascular spaces.
